# Weekday snacking prevalence, frequency, and energy contribution have increased while foods consumed during snacking have shifted among Australian children and adolescents: 1995, 2007 and 2011–12 National Nutrition Surveys

**DOI:** 10.1186/s12937-017-0288-8

**Published:** 2017-10-03

**Authors:** Flávia Fayet-Moore, Véronique Peters, Andrew McConnell, Peter Petocz, Alison L. Eldridge

**Affiliations:** 1 Nutrition Research Australia, Level 13 167 Macquarie St, Sydney, NSW 2000 Australia; 2 Nestlé Research Center, Lausanne, Switzerland; 3 Macquarie University, Sydney, NSW Australia

**Keywords:** Trends, Snack, Snacking, Nutrition survey, Dietary pattern

## Abstract

**Background:**

There are limited data on the evolution of eating habits, including snacking, in Australia. This study aimed to understand snacking trends among Australian children over three previous National Nutrition Surveys.

**Methods:**

Data were analysed from a single weekday 24-h recall in the National Nutrition Surveys 1995, 2007, 2011–12 among children 2-16y (*n* = 8258). A snacking occasion was defined as an eating occasion that occurred between meals based on time of day.

**Results:**

The percentage of children snacking increased over time (92.5 ± 0.5(SE) % in 1995, 98.1 ± 0.3% in 2007, and 95.8 ± 0.4% in 2011–12) (*P* < 0.001), particularly among those having four or more snacking occasions (7.1 ± 0.5% in 1995, 17.9 ± 0.6% in 2007, and 18.5 ± 0.8% in 2011–2) (*P* < 0.001). The mean number of snacking occasions increased from 2.0 ± 0.0 in 1995, to 2.5 ± 0.0 in 2007 and 2011–12 (*P* < 0.001). The energy contribution from snacking increased from 24.1 ± 0.3% in 1995 to 27.7 ± 0.3% in 2007 and 30.5 ± 0.4% in 2011–12 (*P* < 0.001), while the energy from discretionary food during snacking decreased from 56.5 ± 0.7% in 1995 to 47.3 ± 0.5% in 2007 and 47.9 ± 0.7% in 2011–12 (*P* < 0.001). There were differences in the top foods consumed during snacking: non-alcoholic beverages were prominent contributors in 1995 but not in 2007 or 2011, and pome fruit was the second top energy contributor during snacking in 2007 and 2011 but only fourth in 1995.

**Conclusions:**

Snacking is a prominent dietary pattern that has increased over time in frequency and energy contribution. Foods and beverages consumed during snacking occasions include a mix of core foods and discretionary foods, and while the contribution of discretionary foods has decreased, there is still an opportunity to encourage consumption of more nutrient dense foods during snacking.

**Electronic supplementary material:**

The online version of this article (10.1186/s12937-017-0288-8) contains supplementary material, which is available to authorized users.

## Background

The prevalence of overweight and obesity among children and adolescents has risen across the globe [1, 2]. In Australia, in 2014–15 27.4% of children aged 5–17 years were overweight or obese [3], up from 25.7% in 2011–12, 24.7% in 2007, and 20.9% in 1995 [4]. Rates of overweight and obesity have concurrently increased with major shifts in meal patterns, food choices and location of food consumption [5–8]. In the United States (US), snacking has become more prevalent and all snacking combined, contribute as much to total energy intake as a meal [9–12]. In Northern Ireland and Great Britain, both energy and portion size of snacks increased, but not snacking frequency [13]. Snacking is a prevalent eating behaviour in both Mexico [14, 15] and Brazil [16] and has also increased among countries where it is less traditional including China [17, 18] and Spain [19].

There is a long-held concern that increased snacking contributes to obesity [20], but a number of recent reviews [21–23] and a meta-analysis of cross sectional studies and a case-control study, [24] found that eating frequency and snacking were not associated with obesity, whereas a recent cross-sectional study found that only after adjustment for misreporting, snacking frequency was associated with BMI percentile and waist circumference in children, but not in adolescents [25]. However, the concern is that snacking contributes a significant proportion of total energy to children’s diets [10, 11, 14, 16, 18] and increased eating frequency is associated with greater total energy intake [9, 15, 26–28], which may contribute to the childhood obesity epidemic. Further, the type and hence quality of the ‘snack’ choice may influence total energy intake and the association between snacking and obesity [22].

Limited studies report on trends in meal and snacking patterns among children. It is necessary to capture changes in eating behaviour for successful, culturally relevant recommendations and intervention programs, as unhealthy eating patterns during childhood and adolescence can have implications in the development of chronic disease [29, 30] into adulthood. In Australia, dietary guidelines are food-based rather than meal-based. Understanding meal patterns can provide insight for the food-based approach in recommendations with regards to specific timing of food consumption and the distribution of food groups across the day. Given the shifts in dietary patterns worldwide and the rise in childhood obesity, there is a need to evaluate changes in eating patterns among Australian children. The objective of this study is to investigate the changes in snacking patterns in terms of prevalence, energy and nutrient contribution to total daily intakes and food groups consumed among children and adolescents across three nationally representative nutrition surveys in Australia, spanning 16 years.

## Methods

### Comparison of three surveys

Data from three nationally representative nutrition surveys among Australian children and adolescents were used: the 1995 National Nutrition Survey (NNS) [31], the 2007 National Children’s Nutrition and Physical Activity Survey (NCNPAS) [32], and the 2011–12 National Nutrition and Physical Activity Survey (NNPAS) [33]. The 1995 NNS and 2011–12 NNPAS were conducted by the Australian Bureau of Statistics (ABS). The 2007 NCNPAS was carried out by the Commonwealth Scientific and Industrial Research Organisation and the University of South Australia. The 1995 NNS and 2011–12 NNPAS surveyed participants aged 2 years and over; while in the 2007 NCNPAS participants were aged 2–16 years.

For all three surveys, households were selected from all Australian states and territories, across urban and rural dwellings. The 1995 NNS and 2011–12 NNPAS used a stratified multi-stage area sampling plan to identify private households, whereas the 2007 NCNPAS recruited households using Random Digit Dialling. For the 1995 NNS and 2011–12 NNPAS, one adult and one child were randomly selected from each household, while one child only was selected for the 2007 NCNPAS. In the 1995 NNS and 2011–12 NNPAS interviews were conducted throughout the year to account for seasonal variation in intakes, while in the 2007 NCNPAS interviews were conducted between February and August 2007. For all three survey interviews were conducted on all seven days of the week. Data were collected by proxy for younger children, and with the assistance of an adult member of the household for older children. Trained interviewers used the 24-h dietary recall method to collect information on foods and beverages consumed on the day prior to the interview. The 1995 NNS interviewers and the 2007 NCNPAS interviewers used a three-pass method, whereas the 2011–12 NNPAS data were collected with an enhanced five pass method. The 1995 NNS used a ‘3 phase multiple pass’ method developed by the United States Department of Agriculture [31]. The 2007 NCNPAS used a standardized, computer-based, three-pass method which employed the following steps: 1st pass – a ‘quick list’ of all foods and beverages consumed, 2nd pass – time and place of consumption for each item, 3rd pass – a ‘recall review’ for corrections and additions [32]. The 2011–12 NNPAS used the Automated Multiple-Pass Method developed by the Agricultural Research Service of the United States Department of Agriculture [34]. This is a five-pass method which involved the following steps: phase one – ‘quick list’, phase two – ‘forgotten foods’, phase three – ‘time and occasion’, phase four – ‘detail cycle’, phase five – ‘final probe’. Two days of recall were obtained in all three surveys, however, in 1995, only 10% of participants had a second day of recall compared to 57% in 2011–12 and 100% in 2007. More information on the three surveys is summarised in Additional file 1: Table S1.

For comparative purposes, data among children aged 2–16 years in each survey were examined. Due to significant differences in meal patterns between weekdays and weekends [35, 36], weekend day recalls were excluded. Only day 1 data were used in 1995 and day 2 data were used in 2007 and 2011–12 when day 1 was a weekend day. Data were weighted to represent the Australian population according to weightings provided by the Australian Bureau of statistic for each survey. A total of 2340 children and adolescents were included in 1995, 3637 in 2007, and 2281 in 2011–12. The interview components of the 1995 and 2011–12 were conducted under the Census and Statistics Act 1905. Ethics approval was not necessary for those surveys. In 2007, ethics approval was obtained from the National Health and Medical Research Council registered Ethics Committees of the Commonwealth Scientific and Industrial Research Organisation and the University of South Australia.

### Meal and snacking definitions

Recommendations made by Johnson et al. [22] were taken into consideration in the development of an objective definition of snacking. There are several approaches used in the literature to define snacking: snacking based on time of day [13, 37], food-based [38–40] participant-defined snacking [41, 42], meal patterns [38], or methods that incorporate social, physiological or situational cues (e.g. after waking or based on hunger or hormones present) [43]. Although participant-defined eating occasions were available for the 1995 and 2011–12 surveys, they were not captured in the 2007 survey and hence it was not possible to use this measure. Further, we felt it was necessary to develop an objective measure of snacking, where the participant’s perception of what constitutes a snack or meal was removed entirely from the analysis, as ‘snacking’ and ‘snack’ may be interpreted in different ways by different people or within different contexts, snacking is often associated with less healthful food choices and ‘snack foods’ can be classified by participants as both a snack and as a meal (e.g. cheese on toast) [44].

Each food and beverage consumed at the same time of day is reported as a single eating occasion, fully capturing eating patterns as they are consumed. A time of day approach includes all foods and beverages irrespective of energy content or time between eating episodes. This enables all foods and beverages such as diet soft drinks or water, or very low energy foods (i.e. celery sticks) to be further investigated in relation to other components of daily food intake (such as the effect of that eating occasion on subsequent food consumption). It can misclassify specific meals as snacking and snacking as meals, but only when relating to a participant’s perception of the type of eating occasion. Therefore, the time of day approach was utilised to define when meals and snacking occurs. This was determined using previously published methodology using population energy distribution across the day, by plotting daily percent of energy by time of day in 30-min increments [45].

A meal time period began at the first increase in the percent of daily energy and ended in the trough. Main meals were defined as the three largest peaks of energy, and snacking as the three lowest peaks of energy across the day (online Additional file 2: Figure S1). Main meal and snacking times were similar for all three surveys: breakfast occurred between 05.30–09.30 h (09.00 in 2007); morning snack 09.30–11.30 (09.00 in 2007), the midday meal 11.30–14.30, the afternoon snack 14.30–17.00 (17.30 in 2011–12), the evening meal was 17.00–21.30 (17.30 in 2011–12, 21.00 in 2007) and the late night snack 21.30–05.30 (21.00 in 2007). A sensitivity analysis was performed to investigate the effect of different main meal and snacking times for the three surveys. The times given above for the 2011–12 survey were replicated for the 1995 and 2007 surveys, and absolute energy and energy contribution from snacking, prevalence of consumption at each snacking period, frequency of snacking, and number of snacking occasions were calculated. The results were compared with the results using the survey-specific times, and were found to be sufficiently similar to justify the original methodology (Additional file 3: Tables S2 and S3).

An eating occasion was defined as one or more food or beverage items consumed at the same time of day. A participant who reported a glass of orange juice at 8.15 am, then reported toast and jam at 9.00 am had two eating occasions during ‘breakfast’. Eating occasions that occurred during breakfast, midday and evening meals were meals and all eating occasions that occurred between these meals were classified as snacking. A snacking occasion was defined as one or more food or beverage items consumed at the same time of day within a snacking time period. The prevalence of main meals and snacking consumption was estimated. The mean number of snacking occasions as well as the frequency of snacking were assessed by calculating the percentage of children having zero, one, two, three, or four or more snacking occasions per day.

### Dietary intake data

Dietary intake data were analysed for each survey using the survey specific Australian Food Composition Database (AUSNUT) developed by Food Standards Australia New Zealand (FSANZ) [46–48]. The AUSNUT database is survey specific, resulting in a different database for each survey, and slightly different food groups. While daily energy and macronutrient intakes have been previously published [49], we present mean daily energy intake, mean daily energy during snacking, the percent contribution of energy during snacking to total daily intake, and the contribution of carbohydrate, protein, total fat and total sugars to total energy intake during snacking were estimated for each survey.

Discretionary foods are defined by the Australian Dietary Guidelines as foods and beverages not necessary for a healthy diet and are generally high in saturated fat and/or added sugars, added salt or alcohol and low in fibre [50]. The ABS provided coding for discretionary foods in the 2011–12 NNPAS [51]. Using the matching files created by FSANZ, foods from the 1999 AUSNUT database (used for the 1995 NNS) were cross-referenced with 2007 and 2011–13 (used for the 2011–12 NNPAS) to code for discretionary foods [52, 53]. Since there was no matching file for 2007 to 2011–13, a two-step matching approach was used for 2007. When a food group did not have any matching food, the nutrient profile was compared with 2011–13 foods. Foods with similar nutrient content were then grouped together and classified accordingly as discretionary. When a food matched more than one food group in the 1999 or 2007 database, the food was categorised as discretionary if at least one of the 2011–13 foods was discretionary.

In this study, food groups were reported at the sub-major food group classification. There were a total of 107 sub-major food groups in the AUSNUT database in 1999, 111 in 2007, and 132 in 2011–13. The top ten sub-major food groups that reported the most to energy contribution during snacking were reported for each survey. In addition, the mean energy intake of each food group consumed during snacking, the percent of consumers and the mean portion size in grams among consumers of the food group were calculated.

### Anthropometric measures

Anthropometric measures were collected by trained interviewers who took weight, height, and waist circumference measurements for all consenting and able respondents. Weight was measured using digital scales, height was measured using a stadiometer and waist circumference was measured using a metal tape measure. The BMI z-score was calculated using a child’s age, sex, height, weight and the World Health Organization growth reference standards for 2–4 year olds and 5–19 year olds [54, 55]. The standard normal distribution was then calculated for the BMI z-scores and children were categorized as: normal weight (< 85%), at risk for overweight (≥ 85% to <95%), overweight (≥ 95%). In children a waist circumference to height ratio of <0.5 is associated with a low risk of metabolic complications from obesity, whereas a ratio of >0.5 is associated with a higher risk [56]. Therefore, a waist circumference to height ratio of 0.5 was used as a cut-off for waist circumference and risk of metabolic complications.

### Under-reporters

Under-reporting in dietary surveys can selectively affect foods that are reported, but also specific meals and snacks, especially those that are perceived by individuals as less socially desirable (i.e. consumption of junk foods or that skipping breakfast is ‘unhealthy’) than others [57]. Energy intake to basal metabolic rate ratio (EI:BMR) was used to estimate number of under-reporters, i.e. participants with implausibly low energy intakes. In the 2011–12 NNPAS the EI:BMR was calculated by the ABS and provided as part of the survey data; for the 1995 NNS and 2007 NCNPAS we used the same methodology as the ABS to calculate participants’ BMR using age, sex, and weight, with no adjustment for activity levels (Additional file 4: Table S4). The surveys did not provide the requisite data on physical activity level (PAL) so an EI:BMR of 1.55 was used for the calculation of under-reporting cutoffs in line with the ABS recommendations from the Australian Health Survey. Participants were classified as under-reporters based on the Goldberg [58] cut-off limit of 0.9 for EI:BMR, which is the lower 95% confidence limit for a single day of data for a single individual, allowing for day-to-day variation in energy intakes and errors in calculation of EI:BMR. Under-reporting was included as a factor in the analysis, rather than excluding participants with low energy intakes [59]. A more recent analysis of methods to control for under-reporting also suggests that excluding under-reporters may lead to selection bias and inflated associations [60].

### Statistical analysis

The statistical package IBM SPSS Statistics (Version 23) was used for all analyses. Descriptive summaries were calculated for all variables of interest and chi-squared tests were used to determine statistical significance of categorical variables between survey years. ANOVA tables were produced to calculate standard errors of the mean, and post hoc pairwise comparisons using the Bonferroni correction to show pairwise significance between survey years. A general linear model was used to investigate the effect of age (2–3, 4–8, 9–13, 14–16 years), sex, energy intake, survey year and under-reporting on number of snacking occasions per day. The main effects of the factors were included, together with the interaction of age and sex, and age and year. *P*-values <0.001 were taken to indicate statistical significance.

## Results

There was a higher prevalence of at risk for overweight and overweight children in 2007 (32.7%) and 2011–12 (32.2%) compared to 1995 (27.5%) (Table 1), and a significant increase in mean BMI z-scores from 1995 to 2007 (*P <* 0.001). In addition, there was a significant increase in mean waist-to-height ratio, between 2011 and 12 and both 2007 and 1995 (*P* < 0.001).

**Table 1:** Characteristics of children and adolescents 2-16 years from the Australian National Nutrition Surveys 1995, 2007 and 2011–12

Characteristic	National Nutrition Survey									
	1995			2007			2011–12			
	Mean	SE	CI (95%)	Mean	SE	CI (95%)	Mean	SE	CI (95%)	*P-*value
N	2340			3637			2281			
Males (%)	51.7	1.0	49.6–53.7	51.3	1.0	49.6–52.9	49.8	1.0	47.7–51.8	0.39
Under-reporters^*^ (%)	10.7	0.6	8.8–12.5	11.5	0.7	10.0–13.0	16.3	0.8	14.0–18.6	< 0.001
Age group (%)										< 0.001
2-3 years	13.7	0.7	12.3–15.2	12.6	0.7	11.6–13.7	13.6	0.7	12.2–15.0	
4-8 years	34.0	1.0	32.0–35.9	35.0	1.0	33.5–36.6	32.8	1.0	30.8–34.7	
9-13 years	32.4	1.0	30.5–34.3	32.9	1.0	31.4–34.5	35.2	1.0	33.2–37.1	
14-16 years	19.9	0.8	18.3–21.6	19.4	0.8	18.1–20.6	18.5	0.8	16.8–20.1	
BMI z-score (mean SE)	0.47^a^	0.02	0.42–0.51	0.63^b^	0.02	0.59–0.67	0.60^a,b^	0.03	0.55–0.66	< 0.001
Waist:height ratio (mean SE)	0.46^a^	0.00	0.46–0.46	0.47^a^	0.00	0.47–0.47	0.48^b^	0.00	0.48–0.48	< 0.001
Weight status^†^ (%)										< 0.001
Normal weight	72.5	0.9	70.6–74.3	67.3	1.0	65.7–68.8	67.8	1.0	65.7–70.0	
At risk for overweight	14.0	0.7	12.6–15.5	15.3	0.7	14.2–16.5	12.7	0.7	11.2–14.2	
Overweight	13.5	0.7	12.1–14.9	17.4	0.8	16.2–18.6	19.5	0.8	17.7–21.3	
Waist to Height: Ratio^‡^ (%)										< 0.001
< 0.5	77.1	0.9	75.4–78.9	74.3	0.9	72.9–75.7	66.3	1.0	64.1–68.5	
≥ 0.5	22.9	0.9	21.1–24.6	25.7	0.9	24.3–27.1	33.7	1.0	31.5–35.9	

Different superscripts a,b denote significant difference between years (post hoc, Bonferroni, *P* < 0.001).

^*^Participants were classified as under-reporters based on the Goldberg cut-off limit of 0.9 for EI:BMR^(44)^

^†^Calculated using the standard normal distribution of BMI z-scores: normal weight (< 85%), at risk for overweight (≥ 85% to <95%), overweight (≥ 95%)

^‡^In children a waist circumference to height ratio of <0.5 is associated with a low risk of metabolic complications from obesity, whereas a ratio of >0.5 is associated with a higher risk [56]. Therefore, a waist circumference to height ratio of 0.5 was used as a cut-off for waist circumference and risk of metabolic complications

*P*-values for the comparison between the years 1995, 2007 and 2011–12. Chi-square tests were performed for categorical variables and ANOVA for numerical variables

### Snacking prevalence and frequency

There was an increase in the proportion of children snacking, from 92.5% in 1995 to 98.1% in 2007, as well as an increase in the proportion of consumers during each snacking period (morning, afternoon, and late night) (Table 2). From 2007 to 2011–12 there was a slight decline in the percentage of children snacking overall and during each snacking period, however the prevalence was substantially higher in 2011–12 than 1995.

**Table 2:** Snacking pattern of children and adolescents 2-16 years from the Australian National Nutrition Surveys 1995, 2007 and 2011–12

Characteristic	National Nutrition Survey									
	1995 (*n* = 2340)			2007 (*n* = 3637)			2011–12 (*n* = 2281)			*P*-value
Prevalence of consumption at each meal and snacking period^*^	%	SE	CI (95%)	%	SE	CI (95%)	%	SE	CI (95%)	
Breakfast	89.4	0.6	88.1–90.7	95.6	0.3	95.0–96.3	91.5	0.6	90.4–92.7	< 0.001
Morning snack	74.4	0.9	72.6–76.2	90.5	0.5	89.5–91.4	83.1	0.8	81.6–84.7	< 0.001
Midday meal	91.4	0.6	90.2–92.5	94.4	0.4	93.7–95.2	90.0	0.6	88.7–91.2	< 0.001
Afternoon snack	76.1	0.9	74.3–77.8	83.5	0.6	82.3–84.7	82.2	0.8	80.7–83.8	< 0.001
Evening meal	96.6	0.4	95.8–97.3	98.1	0.2	97.6–98.5	94.0	0.5	93.0–95.0	< 0.001
Late night snack	10.3	0.6	9.0–11.5	12.5	0.5	11.4–13.6	9.3	0.6	8.1–10.5	< 0.001
Any meal period	99.9	0.1	99.9–100.0	100	0.0	99.9–100.0	99.8	0.1	99.6–100.0	0.174
Any snacking period	92.5	0.6	91.4–93.6	98.1	0.3	97.7–98.6	95.8	0.5	95.0–96.6	< 0.001
Frequency of snacking										< 0.001
Non-snackers (0 snacking occasions)	7.5	0.5	6.4–8.6	1.9	0.2	1.4–2.3	4.2	0.4	3.4–5.0	
1 snacking occasion	23.6	0.9	21.9–25.4	13.7	0.6	12.6–14.8	15.5	0.8	14.0–17.0	
2 snacking occasions	43.5	1.0	41.4–45.5	41.0	0.8	39.4–42.6	34.9	1.0	33.0–36.9	
3 snacking occasions	18.3	0.8	16.7–19.9	25.5	0.7	24.1–26.9	26.9	0.9	25.1–28.7	
4+ snacking occasions	7.1	0.5	6.1–8.2	17.9	0.6	16.7–19.1	18.5	0.8	16.9–20.1	
	mean	SE	CI (95%)	mean	SE	CI (95%)	mean	SE	CI (95%)	
Number of snacking occasions	2.0 ^a^	0.02	1.9–2.0	2.5 ^b^	0.02	2.5–2.6	2.5^b^	0.03	2.4–2.5	< 0.001
2-3 years	2.2	0.06	2.1–2.3	2.7	0.08	2.6–2.8	2 .6	0.07	2.5–2.8	< 0.001
4-8 years	1.9	0.03	1.8–2.0	2.6	0.04	2.5–2.6	2.6	0.04	2.5–2.7	< 0.001
9-13 years	2.0	0.03	1.9–2.1	2.5	0.05	2.4–2.6	2.5	0.04	2.4–2.6	< 0.001
14-16 years	1.9	0.05	1.7–2.0	2.4	0.06	2.3–2.5	2.2	0.07	2.1–2.3	< 0.001
Total daily energy intake (kJ)	8603^a^	73	8457–8748	8119^b^	49	8023–8216	7756 ^b^	63	7631–7880	< 0.001
Energy during snacking (kJ)	2094^a^	38	2020–2167	2256^b^	26	2205–2307	2366 ^c^	37	2293–2439	< 0.001
Energy contribution of snacking to total daily energy intake (%)	24.1^a^	0.3	23.4–24.8	27.7 ^b^	0.3	27.2–28.2	30.5^a,b^	0.4	29.7–31.3	< 0.001
Contribution from macronutrients to total energy intake during snacking (%)										
Protein	9.4^a^	0.1	9.1–9.6	11.7^b^	0.3	11.2–12.2	11.2^b^	0.1	11.0–11.5	< 0.001
Fat	29.7^a^	0.3	29.1–30.3	28.2^b^	0.2	27.7–28.6	28.4^a,b^	0.3	27.8–29.0	< 0.001
Carbohydrate	62.6^a^	0.4	61.9–63.4	59.8^b^	0.3	59.3–60.3	59.0^b^	0.3	58.3–59.6	< 0.001
Total sugars	38.9^a^	0.5	37.9–39.9	33.8^b^	0.3	33.2–34.4	32.0^b^	0.4	31.2–32.9	< 0.001
Total discretionary^†^ energy intake (kJ)	3680^a^	53	3577–3783	2931^b^	35	2863–2999	2911^b^	46	2820–3001	< 0.001
Contribution of snacking to total discretionary energy (%)	33.6^a^	0.6	32.4–34.7	37.9^b^	0.5	37.0–38.8	40.0 ^b^	0.7	38.7–41.4	< 0.001
Energy from discretionary food during snacking (%)	56.5 ^a^	0.7	55.0–57.9	47.3 ^b^	0.5	46.2–48.4	47.9 ^b^	0.7	46.5–49.4	< 0.001

Different superscripts a,b,c denote significant differences between years (post hoc, Bonferroni, *P* < 0.001)

^*^Meal and snack time periods were defined by time of day: breakfast occurred between 05.30–09.30 h (09.00 in 2007); morning snack 09.30–11.30 (09.00 in 2007), the midday meal 11.30–14.30, the afternoon snack 14.30–17.00 (17.30 in 2011–12), the evening meal was 17.00–21.30 (17.30 in 2011–12, 21.00 in 2007) and the late night snack 21.30–05.30 (21.00 in 2007)

^†^Discretionary foods are defined by the Australian Dietary Guidelines as foods and beverages not necessary for a healthy diet and are generally high in saturated fat and/or added sugars, added salt or alcohol and low in fibre [50]

*P*-values for the comparison between the years 1995, 2007 and 2011–12. Chi-square tests were performed for categorical variables and ANOVA for numerical variables

The frequency of snacking rose across survey years with a higher percentage of children consuming three or four or more snacking occasions in 2007 (25.5% and 17.9% respectively) and 2011–12 (26.9% and 18.5% respectively) compared to 1995 (18.3% and 7.1% respectively) (Table 2). The percentage of children who had fewer than three snacking occasions progressively decreased from 74.6% in 1995 to 56.6% in 2007 and 54.6% in 2011–12.

**Fig. 1:**
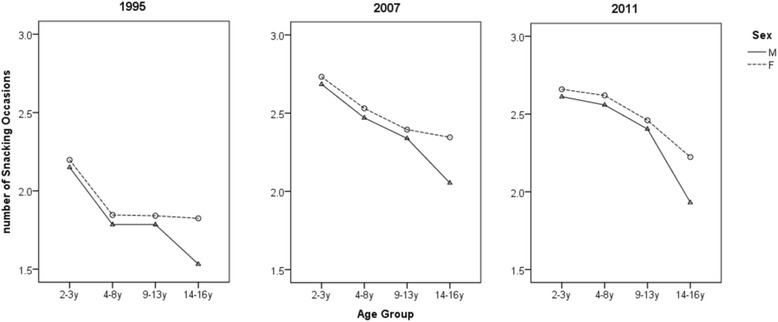
Mean number of snacking occasions by age group, sex, and year, adjusted for energy intake and underreporting. In 1995, for 2-3y *n* = 321, 4-8y *n* = 795, 9-13y *n* = 758, 14-16y *n* = 466. In 2007, for 2-3y *n* = 460, 4-8y *n* = 1275, 9-13y *n* = 1198, 14-16y *n* = 704. In 2011–12, for 2-3y *n* = 310, 4-8y *n* = 748, 9-13y *n* = 802, 14-16y *n* = 421

The mean number of snacking occasions per day increased significantly from 1995 to 2007 and 2011–12 (*P* < 0.001). Figure 1 represents the model for the mean number of snacking occasions with age group, sex, energy intake, year, and under-reporting, all showing statistical significance (*P* < 0.001), however the *R*-squared value is only 8%. Number of snacking occasions was directly proportional to energy intake for all three years investigated, decreased with age and was higher among females than males, though the effect size was quite small. From the figure, the largest sex difference is about 0.3 snacking occasions in 14-16y olds in 1995, the largest age group difference is about 0.7 (between 2-3y and 14-16y boys in 2011), and the largest year difference is about 0.5 (in 14-16y boys between 1995 to 2007). The standardised effect sizes (for sex, age group and year) are between 0.15 and 0.35 and hence, they are ‘small’. The low *R*-squared value implies that the variance in snacking frequency cannot be accurately explained by age, sex and energy intake alone. While the number of snacking occasions increased from 1995 to 2007 and 2011–12, the factors that were significantly associated with frequency of snacking remained the same in each survey year.

### Energy and macronutrients

While total daily energy intake decreased from 1995, energy intake from snacking increased significantly (Table 2). As a result, the contribution of snacking to total energy intake also increased significantly. The contribution from protein to total energy during snacking increased from 1995, but the contributions from fat, carbohydrate, and total sugars all decreased. In addition, the proportion of discretionary energy that came from snacking increased (33.6% vs. 37.9% vs. 40.0%, *P* < 0.001), whereas the proportion of total snacking energy that came from discretionary foods decreased significantly from 1995 to 2011–12 (56.5% vs. 47.9%, *P* < 0.001).

**Fig. 2:**
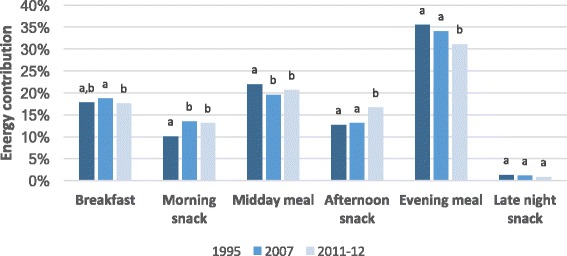
Mean per capita contribution of each eating period to total energy intake among Australian children 2-16y, based on National Nutrition Surveys from 1995, 2007 and 2011–12. Different superscripts a, b denote significant difference (*P* < 0.001, post hoc Bonferroni) between years for the same meal or snack period. In 1995 *n* = 2340, 2007 *n* = 3637, 2011–12 *n* = 2281

Figure 2 shows that in 2007 and 2011–12, energy intake was more evenly distributed throughout the day than in 1995. This pattern is most evident in the middle four meal and snack periods: in 1995 the midday and evening meals contributed 57.5% of total daily energy intake, compared to 51.7% in 2011–12; in 1995 the morning and afternoon snacking periods contributed 22.8% of energy intake compared to 29.7% in 2011–12. In contrast, there was almost no change between 1995 and 2011–12 for breakfast and the late night snack period.

### Food groups consumed during snacking

Top food groups that contributed the most to energy intake during snacking included a combination of those that were predominantly core foods and beverages (bread, dairy milk, pome fruit) and predominantly discretionary (sweet biscuits, cakes, potato snacks) in all three surveys (Table 3) [50]. Beverages were popular in 1995, with fruit and vegetable juices and drinks the top-ranked food group, and soft drinks, flavoured mineral waters and electrolyte drinks the ninth-ranked food group. In contrast, aside from dairy milk, these non-alcoholic beverages did not appear in the top ten in 2007 or 2011–12.

**Table 3:** Top 10 food and beverages by percent energy contribution to snacking among Australian children 2-16y, based on the National Nutrition Surveys 1995, 2007 and 2011–12

Ranking		Food and beverage group^a^		Contribution to total energy from snacking (%)		Consumers during snacking (%) (*n* = 2340)		Energy intake during snacking (kJ)		Portion size per consumer (g)	
		Mean	SE	CI (95%)	%	Mean	SE	CI (95%)	Mean	SE	CI (95%)
1995											
1	Fruit and vegetable juices, and drinks	8.6	0.4	7.8–9.3	34.7	160.7	7.0	147.0–174.4	268.4	9.3	250.0–286.7
2	Sweet biscuits	7.1	0.4	6.4–7.8	22.3	138.2	7.7	123.2–153.2	31.8	1.3	29.3–34.4
3	Regular breads, and rolls	6.8	0.3	6.2–7.4	23.7	181.2	8.5	164.4–197.9	72.1	2.1	68.0–76.1
4	Pome fruit	5.6	0.3	4.9–6.3	22.1	68.2	3.1	62.2–74.3	153.4	3.7	146.1–160.6
5	Dairy milk	5.4	0.3	4.8–6.0	19.0	131.7	7.5	117.0–146.4	271.8	9.9	252.3–291.2
6	Cakes, buns, muffins, scones, cake-type desserts	5.4	0.3	4.7–6.1	12.9	144.5	10.2	124.4–164.5	79.4	3.7	72.0–86.7
7	Chocolate and chocolate-based confectionery	4.1	0.3	3.5–4.6	14.1	92.9	8.0	77.2–108.5	32.9	2.2	28.6–37.2
8	Potato snacks	3.8	0.3	3.3–4.3	11.6	78.3	5.7	67.2–89.5	31.7	1.4	29.0–34.5
9	Soft drinks, flavoured mineral waters and electrolyte drinks	3.5	0.3	2.9–4.0	13.5	73.4	5.2	63.3–83.6	368.7	14.1	341.0–396.3
10	Savoury biscuits	3.3	0.2	2.9–3.8	14.0	63.9	4.5	55.0–72.8	23.6	1.1	21.4–25.7
Ranking		Food and beverage group^a^		Contribution to total energy from snacking (%)		Consumers during snacking (%) (*n* = 3637)		Energy intake during snacking (kJ)		Portion size per consumer (g)	
		Mean	SE	CI (95%)	%	Mean	SE	CI (95%)	Mean	SE	CI (95%)
2007											
1	Sweet biscuits	7.0	0.3	6.5–7.6	23.6	138.7	5.3	128.2–149.1	29.7	0.7	28.3–31.1
2	Pome fruit	6.9	0.3	6.4–7.4	29.8	107.2	3.3	100.8–113.6	158.4	2.6	153.3–163.5
3	Regular breads, and bread rolls	6.3	0.2	5.8–6.7	22.1	162.5	6.2	150.4–174.7	69.7	1.5	66.8–72.6
4	Cakes, buns, muffins, scones, cake-type desserts	6.1	0.3	5.5–6.6	13.9	168.1	8.7	151.0–185.3	80.4	2.5	75.4–85.3
5	Savoury biscuits	5.1	0.2	4.7–5.6	22.8	101.7	4.3	93.4–110.1	23.9	0.6	22.6–25.2
6	Dairy milk (cow, sheep and goat)	4.7	0.2	4.3–5.1	18.2	119.3	5.2	109.2–129.4	251.8	6.1	239.8–263.7
7	Cereal-, fruit-, nut- and seed-bars	3.8	0.2	3.4–4.1	14.5	76.1	3.5	69.2–82.9	32.8	0.7	31.4–34.2
8	Tropical fruit	3.7	0.2	3.3–4.1	15.7	59.6	2.5	54.6–64.6	112.3	2.2	108.0–116.7
9	Potato snacks	3.6	0.2	3.2–4.0	11.9	79.0	4.5	70.3–87.8	30.9	1.0	28.8–32.9
10	Chocolate and chocolate-based confectionery	3.0	0.2	2.6–3.3	10.9	67.2	4.3	58.8–75.6	29.8	1.3	27.3–32.4
Ranking		Food and beverage group^a^		Contribution to total energy from snacking (%)		Consumers during snacking (%) (*n* = 2281)		Energy intake during snacking (kJ)		Portion size per consumer (g)	
		Mean	SE	CI (95%)	%	Mean	SE	CI (95%)	Mean	SE	CI (95%)
2011–12											
1	Sweet biscuits	6.7	0.3	6.1–7.4	23.7	154.8	8.5	138.2–171.5	33.4	1.3	30.8–36.0
2	Pome fruit	6.6	0.3	6.0–7.2	26.3	111.2	4.5	102.4–120.1	176.8	3.8	169.3–184.3
3	Regular breads, and bread rolls	5.8	0.3	5.2–6.3	20.0	136.5	6.4	124.0–149.0	64.6	1.3	62.0–67.2
4	Cakes, muffins, scones, cake-type desserts	5.7	0.4	5.0–6.4	19	192.6	13.6	165.9–219.3	106.8	4.3	98.3–115.2
5	Savoury biscuits	5.1	0.3	4.5–5.7	19	127.0	8.2	110.9–143.0	33.7	1.6	30.6–36.8
6	Mixed dishes where cereal is the major ingredient	4.3	0.3	3.7–4.9	9.2	153.0	12.0	129.5–176.6	238.2	10.7	217.1–259.4
7	Dairy milk (cow, sheep and goat)	4.1	0.3	3.5–4.6	15.3	95.2	5.9	83.6–106.7	244.9	8.7	227.7–262.1
8	Potato snacks	3.6	0.3	3.1–4.1	12.1	87.3	7.6	72.5–102.2	34.1	2.2	29.7–38.5
9	Muesli or cereal style bars	3.3	0.2	2.9–3.7	12.5	69.0	4.2	60.7–77.2	32.6	0.8	31.0–34.3
10	Tropical and subtropical fruit	2.8	0.2	2.4–3.2	12.9	51.1	3.0	45.2–57.0	107.3	2.8	101.7–112.8

^a^ Note: All three surveys contained the food group “fruit and vegetable juices, and drinks”, which included cordials in 1995, but in 2007 and 2011–12 “cordials” was a separate food group. The 1995 food group “soft drinks, flavoured mineral waters and electrolyte drinks” was composed of two food groups in 2007 and 2011: “soft drinks, and flavoured mineral waters” and “electrolyte, energy and fortified drinks”. The 1995 food group “cereal-, fruit-, nut- and seed-bars” was divided into two groups in 2007: “cereal-, fruit-, nut- and seed-bars” and “breakfast cereals and bars, unfortified and fortified varieties”, and two groups in 2011–12: “fruit, nut and seed-bars” and “muesli or cereal style bars”

Among discretionary foods, the sub-major food group cakes, buns, muffins, scones, cake-type desserts appeared in the top ten in all three surveys, but their contribution to snacking energy intake varied from 5.4% in 1995 to 6.1% in 2007 and 5.7% in 2011–12. The portion size for this food group was 79.4 g in 1995 compared to 106.8 g in 2011–12. Although the energy contribution from sweet biscuits and potato snacks remained constant between 1995 and 2011–12, the prevalence of consumption of these food groups during snacking differed (22.3% vs. 23.7%, 11.6% vs. 12.1% respectively) as well as the amount consumed (31.8 g vs. 33.4 g, 31.7 g vs. 34.1 g respectively). Energy contribution from chocolate and chocolate-based confectionery to snacking was 4.1% in 1995, 3.0% in 2007, and was not in the top ten in 2011–12.

Among core foods, the contribution from dairy milk and regular breads to energy intake during snacking varied between 1995 and 2011–12 (19% vs. 15.3%, 23.7% vs. 20% respectively) due to a reduction in both portion size and prevalence of consumption. Pome fruit, however, showed the opposite trend in proportion of energy intake from 5.6% in 1995, to 6.9% in 2007 and 6.6% in 2011–12. This was due to changes in prevalence of consumption (22.1% in 1995, 29.8% in 2007) and portion size (153.4 g in 1995, 176.8 g in 2011–12).

## Discussion

This study investigated trends in snacking patterns among Australian children and adolescents using three nationally representative surveys. It showed that dietary patterns have shifted to include a greater prevalence and frequency of snacking, with more than double the number of children in 2011–12 having four or more snacking occasions per day than in 1995. These findings are consistent with those in both developing and developed countries [8–12, 16, 18, 19, 21, 43, 61, 62]. Over three decades in the US, the prevalence of snacking increased from 74% in 1977–78 to 98% in 2003–06, while the prevalence of children with less frequent snacking (one or two snacking occasions per day) decreased from 26% to 20% [11]. Similarly in China, where a snacking eating pattern is less prevalent, the proportion of children who snacked between 2004 and 2006 more than doubled [18].

In the present study, the energy contribution from snacking in Australian children increased from 24% in 1995 to 30.5% in 2011–12. Similarly, snacking contributes over a quarter of total energy intake in the US [10, 11], 21% in Scotland [43], 23.3% in Brazil [16], and 32.5% in the United Kingdom [13], but less than 20% in other countries such as Russia and the Philippines [5], Mexico [14, 15] and China [18]. Thus, this highlights the importance of having culture-specific snacking recommendations.

With the greater proportion of daily energy from snacking, we showed a concurrent decrease in energy contribution from main meals. Over the three surveys, the peaks in energy distribution across the day ‘flattened’. This has major implications for school-aged children in meeting their increased nutrient needs [63], as more food is now being consumed outside of traditionally nutrient-rich main meals, and hence, these main meals are contributing less to total energy intake and possibly to nutrient intakes. We found that the greatest change in energy distribution occurred in meals and snacks in the middle of the day. Breakfast and the late night snack, which among children are traditionally consumed at home rather than away from home, remained constant. It was not possible to report on foods consumed at home or away from home in Australia because location of food consumption was only reported in the 2007 survey. In other countries, food consumed away from home is on the rise [5–7, 64–67]. Several factors may play a role in changing meal patterns, including increased incomes and the ability to purchase more foods and beverages outside the home [68, 69], fewer family meals at home, dual-working parents [70], urbanisation [71, 72] and increased food availability and affordability [73].

The clear trend of a decrease in snacking with age across all three surveys in Australia is also reported worldwide [5, 10, 11, 14] with the greatest increase in snacking prevalence observed among the younger age groups [10, 14]. In our model, total energy intake was the second most important determinant of number of snacking occasions in all three survey periods. Studies report a positive association between frequency of eating occasions and energy intake [15, 27, 74, 75], but others report that snacking frequency may also assist with energy regulation and that snacking is not necessarily associated with poor diet quality [21]. Our study showed that the relationship between snacking and diet quality is modulated by food choice during snacking, its frequency and portion size. The contribution of snacking to total discretionary energy increased 2011–12 compared to 1995, which was primarily due to the increase in the contribution of snacking to total energy. Main meals contributed the most to discretionary energy intake across all years and the proportion of snacking energy that was discretionary actually decreased. Total discretionary energy intake among children in Australia has declined between 1995 and 2007 [76, 77], and our present findings showed that it continued to decline in 2011–12. Despite snacking contributing less to total discretionary intake, it still represents just under half of discretionary energy intake. Thus an overall reduction in discretionary intake is still warranted, from both meals and snacking. Our findings imply that practical dietary recommendations around snacking need to be culturally relevant and age-appropriate with a focus on the nutrient density or quality of the snack. Under-reporting has increased between 1995 and 2011, and adjustment does not fully account for the fact that certain ‘less-healthy’ foods or beverages may be more likely to be under-reported than others [78]. Thus, true differences in foods consumed across the three surveys may be due to underreporting alone.

We found that a mixture of traditional ‘snack foods’ (i.e. sweet biscuits, and fresh fruit) and ‘meal’ foods (i.e. bread and milk) were consumed during snacking in Australia. Non-alcoholic beverages (fruit and vegetable juices and soft drinks) were among the top contributors of snacking energy in 1995 but did not feature in the top ten in 2007 or 2011–12; pome fruit was the fourth-highest contributor in 1995 and the only fruit in the top ten in 1995, whereas it was the second-highest contributor in 2007 and 2011–12, and tropical fruits also appeared in the top ten. Cakes and desserts remained a top contributor to snacking energy in all surveys, in line with previous Australian reports on trends in food groups consumed [76, 77, 79]. Varying trends in foods and beverages consumed during snacking are reported among children and adolescents and seem to be country-specific. They include a mixture of both core and discretionary snack foods and beverages. For example, fruit, milk and sugar-sweetened beverages as snacks have increased in China [18]. In contrast, the percentage of fruit and milk consumed as a snack decreased in the US between 1977 and 78 and 2003–06 [11], but the energy density of snacks remained the same. A more recent US study, using nationally representative nutrition data from 2003 to 2010, also reported a decrease in energy from discretionary foods among children of all weight categories aged 2–5 years and 12–19 years, but not among those overweight aged 6–11 years [80]. In contrast, in the Philippines, milk and soft drinks consumed as snacks more than doubled between 1994 and 2002 while fast food intake did not change [5]. The effect that snacking has on children’s diet quality is therefore dependent on food choice. In a sub-sample of American children, weekday self-reported snacking was positively associated with age-adjusted diet quality among 9–11 year olds and negatively associated with diet quality in those 12–18 years old [28], but foods consumed as snacks were not reported in that study. Among British adolescents, a positive relationship existed between diet quality and number of snacks per day but this was dependent on the nutritional quality of the snack food or beverage consumed [75]. A recent study among British children and adolescents reported that snack frequency was associated with a lower diet quality score [81]. Similarly, meal frequency has been associated with higher diet quality among children, but the association of snacking frequency with diet quality was dependent on the definition used: time of day or energy contribution [82].

As a consequence of the mixture of nutrient-dense and nutrient-poor food choices during snacking, emphasis on public health and nutrition intervention programs should encourage nutritious snack food choices based on the core food groups, especially since a snacking dietary pattern has become more prevalent. For example, in Australia, the primary school in-class Crunch&Sip® program was introduced in 2005 by the government to encourage students to consume fruit or vegetables and water during class and develop healthy eating behaviours. Nearly half of surveyed government schools have implemented this program into >80% of classrooms [83] and perhaps the increase in fruit intake that was observed on weekdays during snacking in the present study could be, in part, due to this program. It is especially important to focus on the provision of foods and beverages to children, as when given the choice between less nutrient dense and more nutrient dense snack options, children tend to choose the less nutrient dense one: they choose sugar-sweetened beverages over fresh fruit [84].

The strengths of our study include the use of an objective definition of snacking based on time-of-day. This removes any participant bias for what constitutes a ‘snack food’. Even among children’s care-givers, snacks can be defined by type of food, portion size, time of day, eating episode, location, or even purpose [85–87]. A study on adults, by Leech et al. revealed that methodologies based on participants’ self-report of the eating occasions and time-of-day lead to significant differences in snacking frequency but non-significant differences in eating frequency, energy intake and energy contribution from snacking [88]. Further research is needed in order to reach to a consensus on an appropriate definition of meals and snacks [25, 88]. Furthermore, only weekdays were included for consistency in meal patterns, as evidence has shown that weekend patterns are quite different from weekdays [35, 36, 89]. Lastly, prevalence of under-reporting across the three years was accounted for in the general linear model for number of snacking occasions model rather than by excluding those participants from the analysis, which has recently been shown to increase results bias [60].

The present study displays several limitations. Although the trend analysis was based on three nationally representative surveys based on one 24-h dietary recall, the methodology of the recall collection was different across surveys and included both face-to-face (1995 and 2011–12) and telephone interviews (2007). However, results from the US show little difference between in-person and telephone interviews using the automated multiple pass method [34], so we would expect this to have a minimal impact on our findings. The eating pattern analysis was derived from one weekday 24-h recall which is not necessarily representative of usual intakes. In addition, the 2007 survey did not collect data in all months of the year, which may miss differences if snacking patterns in September to January are different from those in the survey months of February through August. It is possible that snacking patterns could differ according to whether the dietary recall day was a school day, but the surveys did not collect data specifying this. Furthermore, the coding of discretionary foods was limited by some differences in food groups across surveys. A systematic approach was used to code for discretionary foods across the surveys in order to minimise bias and accurately capture changes in food group intakes. A limitation to the time of day definition approach is that the time of eating may have changed over the time period studied in response to external influences (e.g. differences in school schedules), which may affect the way the consumption peaks are distributed in each survey and therefore the timing used to define snacking. This effect was evaluated in our sensitivity analysis and found to be minimal. Further, by classifying meals and snacking on the basis of aggregated energy intake data is unlikely to detect important between-subject differences or subpopulations who consume meals or who snack at unconventional times of the day.

Lastly, the present study is based on three cross-sectional surveys, which does not enable us to establish any causal link between snacking and obesity.

## Conclusions

In conclusion, in this study investigating trends in weekday snacking across three nationally representative surveys 1995, 2007 and 2011–12, a large proportion of Australian children and adolescents were found to be snackers, with increases in snacking frequency and the contribution of snacking to total energy, but a decrease in the contribution of snacking to discretionary energy. Both discretionary and core foods were consumed during snacking, highlighting the need for targeted snacking recommendations specific to the local culture. Further research to profile timing and reasons for foods consumed during snacking, which include both nutrient dense and nutrient poor choices, will help understand shifts in dietary patterns and nutrient intakes over time and advise health promotion strategies on population-based food and beverage recommendations that are culturally relevant.

## Additional files

Additional file 1: Table S1. Characteristics of the National Nutrition Surveys 1995, 2007 and 2011–12. (DOCX 14 kb)

Additional file 2: Figure S1. Percent of energy consumed by time of day and classification of meal and snack time periods: 1995, 2007 and 2011–12 National Nutrition Surveys. In 1995 *n* = 2340, 2007 *n* = 3637, 2011–12 *n* = 2281. (PDF 71 kb)

Additional file 3: Table S2–S3. Sensitivity analysis: snacking patterns of children and adolescents 2-16y from the Australian National Nutrition Surveys 1995, 2007 and 2011–12. (DOCX 15 kb)

Additional file 4: Table S4. Basal Metabolic Rate (BMR) for each age and sex group. (DOCX 12 kb)

## Abbreviations

ABS: Australian Bureau of Statistics
AHS: Australian Health Survey
AUSNUT: Australian Food Composition Database
BMI: Body Mass Index
EI:BMR: Energy intake to basal metabolic rate ratio
FSANZ: Food Standards Australia New Zealand
NCNPAS: 2007 National Children’s Nutrition and Physical Activity Survey
NNPAS: 2011–12 National Nutrition and Physical Activity Survey
NNS: National Nutrition Survey
US: United States
