# Supplementary material for: Weekday snacking prevalence, frequency, and energy contribution have increased while foods consumed during snacking have shifted among Australian children and adolescents: 1995, 2007 and 2011–12 National Nutrition Surveys
Source: Nutr J. 2017 Oct 3;16:65. doi: 10.1186/s12937-017-0288-8 (PMC5627470; doi:10.1186/s12937-017-0288-8)
Supplement: Supplementary file 1 — Characteristics of the National Nutrition Surveys 1995, 2007 and 2011–12. (DOCX 14 kb) [file 12937_2017_288_MOESM1_ESM.docx]

**Additional file 1: Table S1.** Characteristics of the National Nutrition Surveys 1995, 2007 and 2011-12.

| **Survey Characteristic** | **National Nutrition Survey** | | |
| --- | --- | --- | --- |
|  | **1995** | **2007** | **2011-12** |
| **Day 1 sample size** | 13858 (2+y) | 4487 (2-16y) | 12153 (2+y) |
| **Response rates** | 61.4% | 60.9% | 77.0% |
| **Day 1 sample size**  **(2-16y)** | 2729 | 4487 | 2548 |
| **Sample size used (weekday recall**  **only, 2-16y)** | 2340 (day 1) | 3637 (2730 day 1, 907 day 2) | 2281 (2033 day 1, 248 day 2) |
| **Sample design** | Sub-set of NHS sample, participation was voluntary (private dwellings only). For NHS a stratified multi-stage area sample was used to select households at random. | A quota sampling scheme based primarily on postcodes was used. Households from selected postcodes were selected using Random Digit Dialing. | Sub-set of the AHS, participation was not voluntary. For the AHS a stratified multi-stage area sample was used to select households at random. |
| **Geographical coverage** | Respondents from urban and rural areas in all states and territories were included. Some persons from what would now be known as Very Remote Areas were also included. | Respondents from urban and rural areas in all states and territories were included. Persons from remote areas were also included, but not from Very Remote Areas. | Respondents from urban and rural areas in all states and territories were included. Persons from remote areas were also included, but not from Very Remote Areas. |
| **Weekdays and seasons of data collection** | February 1995 – March 1996  Summer – 21%  Autumn – 29%  Winter – 26%  Spring – 24%  Monday – 18%  Tuesday – 19%  Wednesday – 20%  Thursday – 17%  Friday – 13%  Saturday – 8%  Sunday – 6% | February 2007 – August 2007  Summer – < 1%  Autumn – 48%  Winter – 52%  Spring – 0%  Monday – 15%  Tuesday – 14%  Wednesday – 17%  Thursday – 15%  Friday – 15%  Saturday – 16%  Sunday – 9% | May 2011 – June 2012  Summer – 27%  Autumn – 31%  Winter – 23%  Spring – 19%  Monday – 18%  Tuesday – 18%  Wednesday – 18%  Thursday – 17%  Friday – 14%  Saturday – 12%  Sunday – 4% |
| **Interview method** | Face-to-face pen and paper interviews. | Day 1: Computer Assisted Personal Interviews (CAPI). Day 2: Computer Assisted Telephone Interviews (CATI). | Day 1: Computer Assisted Personal Interviews (CAPI). Day 2: Computer Assisted Telephone Interviews (CATI). |
| **Who conducted the interview** | Nutritionists trained as interviewers for this survey. | Interviewers who had a tertiary degree or higher in dietetics, nutrition, health science, or other relevant disciplines. | Trained and experienced ABS interviewers. |
| **Who completed the interview** | For children 2-4y, the person responsible for preparing the child’s meals. Children 5-11y provided their own dietary recall with the assistance of an adult member of the household. Children 12-16y provided their own dietary recall. | Proxy interviews were conducted for children 2-8y by the primary caregiver. Children aged 9-16y provided their own dietary recall, with consent from the primary caregiver or a responsible adult for children 9-13y. | An adult was interviewed on the child’s behalf for all children 2-14y, however children aged 6-14y were invited to also participate in the interview. Selected children aged 15-16y were personally interviewed with parental consent. |
