# Supplementary figures and images for: Weekday snacking prevalence, frequency, and energy contribution have increased while foods consumed during snacking have shifted among Australian children and adolescents: 1995, 2007 and 2011–12 National Nutrition Surveys
Source: Nutr J. 2017 Oct 3;16:65. doi: 10.1186/s12937-017-0288-8 (PMC5627470; doi:10.1186/s12937-017-0288-8)

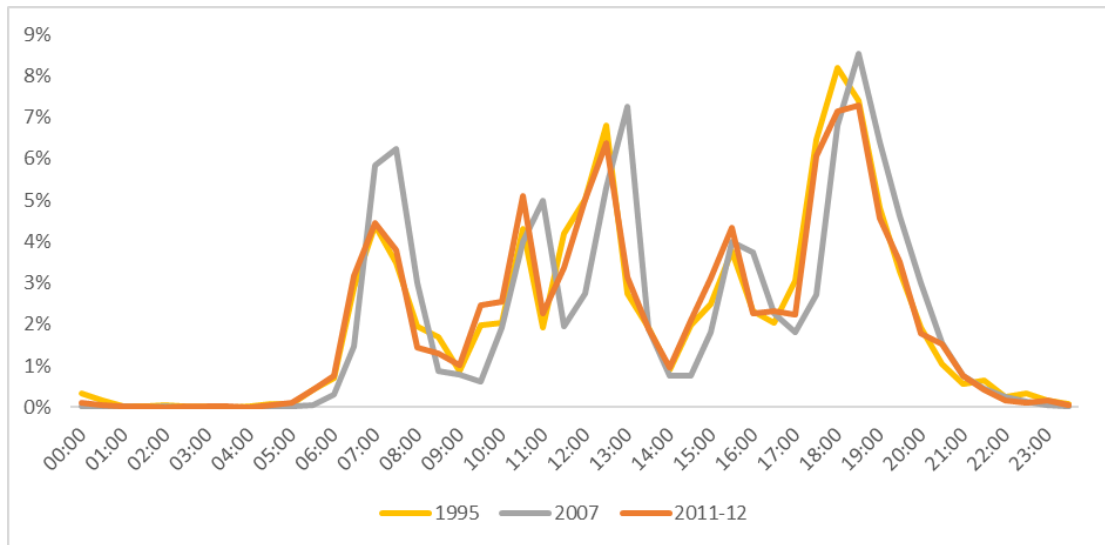

Supplement: Supplementary file 2 — Percent of energy consumed by time of day and classification of meal and snack time periods: 1995, 2007 and 2011–12 National Nutrition Surveys. In 1995 n = 2340, 2007 n = 3637, 2011–12 n = 2281. (PDF 71 kb) [file 12937_2017_288_MOESM2_ESM.pdf]
