# Supplementary material for: Weekday snacking prevalence, frequency, and energy contribution have increased while foods consumed during snacking have shifted among Australian children and adolescents: 1995, 2007 and 2011–12 National Nutrition Surveys
Source: Nutr J. 2017 Oct 3;16:65. doi: 10.1186/s12937-017-0288-8 (PMC5627470; doi:10.1186/s12937-017-0288-8)
Supplement: Supplementary file 3 — Sensitivity analysis: snacking patterns of children and adolescents 2-16y from the Australian National Nutrition Surveys 1995, 2007 and 2011–12. (DOCX 15 kb) [file 12937_2017_288_MOESM3_ESM.docx]

**Additional file 3: Table S2 and Table S3:** Sensitivity analysis: snacking patterns of children and adolescents 2-16y from the Australian National Nutrition Surveys 1995, 2007 and 2011-12*.*

| **Characteristic** | **Original result^+^** | | | **Sensitivity analysis^+^** | | |
| --- | --- | --- | --- | --- | --- | --- |
|  | **1995** | **2007** | **2011-12** | **1995** | **2007** | **2011-12** |
| **Energy during snacking (kJ)** | 2094 | 2256 | 2366 | 2371 | 2353 | 2366 |
| **Energy contribution of snacking to total daily energy intake (%)** | 24.1% | 27.7% | 30.5% | 27.2% | 28.9% | 30.5% |
| **Prevalence of consumption at each snacking period** |  |  |  |  |  |  |
| Morning snack | 74.4% | 90.5% | 83.1% | 74.4% | 87.9% | 83.1% |
| Afternoon snack | 76.1% | 83.5% | 82.2% | 81.2% | 86.6% | 82.2% |
| Late night snack | 10.3% | 12.5% | 9.3% | 10.3% | 8.1% | 9.3% |
| Any snacking period | 92.5% | 98.1% | 95.8% | 94.0% | 97.9% | 95.8% |
| **Frequency of snacking** |  |  |  |  |  |  |
| Non-snackers (0 snacking occasions) | 7.5% | 1.9% | 4.2% | 6.0% | 2.1% | 4.2% |
| 1 snacking occasion | 23.6% | 13.7% | 15.5% | 20.6% | 13.8% | 15.5% |
| 2 snacking occasions | 43.5% | 41.0% | 34.9% | 41.9% | 39.3% | 34.9% |
| 3 snacking occasions | 18.3% | 25.5% | 26.9% | 21.7% | 26.3% | 26.9% |
| 4+ snacking occasions | 7.1% | 17.9% | 18.5% | 9.7% | 18.5% | 18.5% |
| **Number of snacking occasions** | 2.0^a^ | 2.5^b^ | 2.5^b^ | 2.1^c^ | 2.5^d^ | 2.5^d^ |

Different superscripts a,b and c, d denote significant differences between years within the original analysis and the sensitivity analysis (post hoc, Bonferroni, *P*<0.001)

^+^Meal and snack time periods were defined by time of day: breakfast occurred between 05.30 – 09.30 hours (09.00 in 2007); morning snack 09.30 – 11.30 (09.00 in 2007), the midday meal 11.30 – 14.30, the afternoon snack 14.30 – 17.00 (17.30 in 2011-12), the evening meal was 17.00 – 21.30 (17.30 in 2011-12, 21.00 in 2007) and the late night snack 21.30 – 05.30 (21.00 in 2007).

^++^ Meal and snack time periods were defined by time of day: breakfast occurred between 05.30 – 09.30 hours; morning snack 09.30 – 11.30, the midday meal 11.30 – 14.30, the afternoon snack 14.30 – 17.30, the evening meal was 17.30 – 21.30, and the late night snack 21.30 – 05.30.
