# Supplementary material for: Weekday snacking prevalence, frequency, and energy contribution have increased while foods consumed during snacking have shifted among Australian children and adolescents: 1995, 2007 and 2011–12 National Nutrition Surveys
Source: Nutr J. 2017 Oct 3;16:65. doi: 10.1186/s12937-017-0288-8 (PMC5627470; doi:10.1186/s12937-017-0288-8)
Supplement: Supplementary file 4 — Basal Metabolic Rate (BMR) for each age and sex group. (DOCX 12 kb) [file 12937_2017_288_MOESM4_ESM.docx]

**Additional file 4: Table S4.** Basal Metabolic Rate (BMR) for each age and sex group

| **Age group** | **Sex** | **BMR formula** |
| --- | --- | --- |
| 2-3y | Male | 249 * weight (kg) – 127 |
|  | Female | 244 * weight (kg) – 130 |
| 3-9y | Male | 95 * weight (kg) + 2110 |
|  | Female | 85 * weight (kg) + 2033 |
| 10-16y | Male | 74 * weight (kg) + 2754 |
|  | Female | 56 * weight (kg) + 2898 |
